# Bone marrow CD14^+^CD206^+^ M2-like monocyte-macrophages as a potential complementary biomarker for bone marrow MRD in high-risk neuroblastoma

**DOI:** 10.3389/fonc.2026.1904750

**Published:** 2026-10-28

**Authors:** Xiaoyan Hu, Lin Liu, Yuge Ni, Chen Huang, Yan Jin, Baocheng Gong, Tiantian She, Chong Chen

**Affiliations:** 1 Department of Clinical Laboratory, Tianjin Union Medical Center, Nankai University, Tianjin, China; 2 Department of Pediatric Oncology, Tianjin Medical University Cancer Institute and Hospital, National Clinical Research Center for Cancer, Key Laboratory of Cancer Prevention and Therapy, Tianjin’s Clinical Research Center for Cancer, Tianjin, China; 3 School of Medical Laboratory, Tianjin Medical University, Tianjin, China

**Keywords:** biomarker, CD14+CD206+ M2−like macrophage, GD2, minimal residual disease, neuroblastoma

## Abstract

Neuroblastoma (NB) is a highly aggressive pediatric malignancy, frequently associated with a substantial risk of bone marrow metastasis and recurrence. The persistence of minimal residual disease (MRD) remains the primary obstacle to successful treatment outcomes. Bone marrow minimal residual disease (MRD) assessment in high-risk neuroblastoma primarily relies on the direct detection of residual tumor cells. However, the accompanying immune-microenvironmental changes remain incompletely characterized. In this study, GD2-directed immunocytochemistry, complemented by GD2-based flow cytometry, was used for the reference classification of bone marrow disease status in a cohort of 143 patients with high-risk neuroblastoma. We evaluated a panel of peripheral blood biomarkers, identifying NSE, CRP, FBG, and D-dimer as exhibiting significant differences between MRD-positive and MRD-negative groups; however, these biomarkers demonstrated limited diagnostic utility, with area under the curve (AUC) values below 0.7. Importantly, bone marrow CD14^+^CD206^+^ M2-like monocyte-macrophages showed discriminatory ability for GD2-defined bone marrow MRD status (AUC = 0.794), with an optimal threshold of 1.71×10^4^/mL. Additionally, the proportion of CD14^+^CD206^+^ macrophages showed a positive correlation with GD2 positivity in bone marrow samples. These findings suggest that bone marrow CD14^+^CD206^+^ M2-like monocyte-macrophages showed potential as an auxiliary immune biomarker associated with bone marrow MRD status in high-risk NB. These findings require validation in larger, independent cohorts.

## Introduction

Neuroblastoma (NB) originates from the sympathetic ganglia or bilateral adrenal glands, and the long-term survival rate of children with NB in the high-risk group is still less than 50%. The main reasons for this are the susceptibility to bone marrow metastasis and recurrence due to the presence of many microscopic lesions remaining in the bone marrow (1–3). A precise diagnosis of bone marrow-associated NB is of paramount importance in the early detection of metastasis and recurrence in high-risk paediatric NB patients. High-risk patients receive high-intensity chemotherapy, radiotherapy, surgery, and stem cell transplantation with poor clinical prognosis. Some NB patients receive a combination of anti-GD2 antibodies and immunotherapy, as well as 13-cis retinoic acid maintenance to eliminate the microscopic residual disease (MRD), with a more pronounced clinical prognosis (1). High-risk NB recurrence favors the bone marrow and is strongly associated with bone marrow MRD. The current commonly used clinical bone marrow MRD diagnostic technique, bone marrow smear classification technique, has problems such as low sensitivity, poor specificity, lack of quantifiable indexes, and the influence of human factors. These problems make accurate diagnosis difficult. Early monitoring and diagnosis of NB recurrence help early clinical intervention in children with high-risk NB, while problems such as poor diagnostic specificity of peripheral blood NSE test for recurrence diagnosis by the existing testing technology do not allow early warning of MRD recurrence (4). Preclinical findings suggest that NB bone marrow metastasis is closely related to residual GD2-positive cells. GD2 is an important target to solve the challenge of NB bone marrow metastasis, and GD2 results are important laboratory evidence for accurate diagnosis of clinical bone marrow MRD.

Contemporary systematic reviews support a multimodal treatment framework incorporating intensive chemotherapy, consolidation strategies, and GD2-directed immunotherapy in highrisk neuroblastoma. Recent anti-GD2 and GD2-CAR-T studies further illustrate the therapeutic relevance of this antigen, although treatment efficacy does not establish the diagnostic performance of a GD2-based bone marrow assay (4–9).

Recent reviews have emphasized that assessment of low-level neuroblastoma involvement in bone marrow requires sensitive cell-based and molecular approaches because conventional morphology may miss sparse disseminated tumor cells. Immunocytology, multiparametric flow cytometry, and transcript-based assays provide complementary perspectives, although their clinical interpretation depends on specimen type and assessment context (10, 11).

Available clinical biomarkers for laboratory testing of high-risk NB are LDH, NSE, and Ferr. Studies have reported that most neural crest tumors secrete catecholamines (1–3). LDH levels are usually normal in the early stages of the disease and increase significantly with increasing tumor malignancy. Therefore, serum LDH expression levels can be used as a proxy for real-time monitoring of tumor load (12). NSE is a glycolytic enzyme produced in nerve tissue, but NSE levels are also increased in neuroendocrine tumors, so NSE is not specific to NB. They can only be used in patients with a confirmed diagnosis to provide prognostic information and cannot be used in the diagnosis of bone marrow MRD. NB cell lines and tumors produce and secrete glycosylated ferritin (Ferr), which can be distinguished from the non-glycosylated form secreted by healthy cells. Elevated serum Ferr levels have been observed in patients with stage 3 or 4 NB, which is a poor prognostic biomarker for diagnosis and disease stage independent of age. NB is one of the few pediatric malignancies for which biomarkers are routinely used for diagnosis, prediction, and therapeutic monitoring, but few studies have examined the correlation of conventional tumor markers with bone marrow MRD in high-risk NB.

A large number of immunosuppressive cytokines in the tumor microenvironment regulate macrophage phenotype and thus influence their function. There are a large number of immune cells in the microenvironment that can secrete cytokines such as dendritic cells. Myeloid suppressor cells secrete interferon-γ, which induces changes in macrophage phenotype and makes macrophages pro-cancer. The body’s immune response to antitumor effects, cell death occurs in some tumor cells, and dead tumor cells produce factors that can affect the phenotype of macrophages. Tumor metabolism is dramatically abnormal, releasing high levels of lactic acid to produce a specific “Warburg” effect. The metabolic signaling of the “Warburg” effect in tumors directly alters macrophage phenotype and function. M2-like macrophages are closely associated with tumor malignant progression (13). The involvement of bone marrow M2-like macrophages in the immunosuppressive microenvironment and whether they are involved in high-risk NB bone marrow MRD have not been reported to relevant authorities. Recent single-cell studies of neuroblastoma-involved bone marrow have identified remodeling of myeloid and other immune compartments, including macrophage-associated programs and distinct tumor-associated macrophage populations. These findings support investigating marrow monocyte-macrophage phenotypes as correlates of tumor involvement,

without establishing CD206 as a tumor-specific marker (14, 15).

More recent single-cell and multiomic analyses have resolved diverse neuroblastomaassociated myeloid and macrophage states, including treatment-associated changes in the tumor microenvironment. These observations support investigation of macrophage-associated phenotypes but should not be interpreted as direct evidence that CD14^+^CD206^+^ cells are functionally immunosuppressive in the present untreated bone marrow samples (16–19).

We have established GD2-targeted immunocytochemical staining and GD2-based flow cytometry for the detection of bone marrow MRD in high-risk neuroblastoma. These approaches provide direct evidence of residual neuroblastoma cells and serve as important reference methods for MRD assessment. However, direct tumor-cell detection alone does not fully characterize the accompanying changes in the bone marrow immune microenvironment. We therefore sought to determine whether bone marrow immune-cell phenotypes could provide complementary information associated with MRD status. In particular, we focused on monocyte-macrophage subsets that may reflect tumor-associated remodeling of the marrow microenvironment. In pediatric patients, bone marrow sampling itself may also be clinically challenging, further highlighting the need to maximize the information obtained from each available specimen. To solve the dilemma of difficult diagnosis faced by the clinic, this study screened high-risk NB bone marrow MRD-related biomarkers from serum biomarkers and biomarkers in bone marrow aspiration fluid based on the study reference classification of GD2 target detection.

## Methods and materials

### Patients and samples

A total of 143 newly diagnosed pediatric patients with high-risk neuroblastoma aged 2–14 years admitted to the Department of Pediatric Oncology, Tianjin Medical University Cancer Institute and Hospital between January 2020 and March 2023 were enrolled in this study. Inclusion criteria: (1) Histopathologically confirmed neuroblastoma stratified to the high-risk group per the International Neuroblastoma Risk Group system (20); (2) Bone marrow samples collected at initial diagnosis without prior chemotherapy, radiotherapy, surgery or targeted therapy; (3) Complete clinical data and qualified bone marrow specimens; (4) Written informed consent obtained from guardians.

Exclusion criteria: (1) Concurrent other malignancies, autoimmune diseases or severe infections; (2) Extensive bone marrow metastasis with tumor cell proportion ≥5% under morphological examination; (3) Hemolyzed, degraded or undetectable specimens.

Bone marrow samples were harvested from all patients before anti-tumor treatment at diagnosis. A laboratory-developed GD2-targeted combined assay platform was used to assess bone marrow minimal residual disease (MRD). There were 54 MRD-positive cases and 89 MRD-negative cases.

This study was implemented in accordance with the Declaration of Helsinki. Written informed consent was signed by all guardians, and the protocol was approved by the Medical Ethics Committee of Tianjin Medical University Cancer Institute and Hospital (Approval No. BC2022216). Comprehensive genomic and clinical risk-factor data, including MYCN amplification status, 11q and 1p chromosomal abnormalities, image-defined risk factors (IDRFs), and primary tumor site, were not systematically available for the exploratory flow-cytometry subgroup. Therefore, these variables were not included in the present statistical analyses.

### Bone marrow minimal disease assessment and reference classification

Bone marrow samples were obtained at initial diagnosis before the initiation of antitumor therapy. Conventional bone marrow cytomorphology was performed as part of routine clinical assessment. Patients with extensive bone marrow involvement, defined in the present study as a morphologic tumor-cell proportion ≥5%, were excluded in order to focus the analysis on low-level bone marrow disease. This 5% threshold was used solely as a study-specific exclusion criterion and was not intended to represent an INRG diagnostic cutoff for minimal disease.

Published international response and bone marrow reporting frameworks distinguish marrowcytology/immunocytology from imaging-based evaluation of systemic disease and emphasize standardized specimen assessment. These recommendations inform the clinical context of our laboratory reference classification; however, we did not apply a prospectively standardized multicenter response-assessment algorithm or reclassify the baseline samples using treatmentresponse criteria (21–23).

The assessment of low-level bone marrow disease was based on GD2-directed detection of neuroblastoma cells, with reference to the recommendations of the International Neuroblastoma Risk Group (INRG) Task Force for sensitive detection of minimal neuroblastoma cells. GD2-directed immunocytochemistry was used as the principal cell-based reference assessment, while GD2-based flow cytometry was used as a complementary detection method according to our institutional laboratory workflow.

In the present study, GD2-positive neuroblastoma cells were identified according to predefined laboratory interpretation criteria, including specific GD2 staining together with compatible neuroblastoma cell morphology. Bone marrow samples containing unequivocally identified GD2-positive neuroblastoma cells were classified as positive for low-level marrow disease, whereas samples without detectable GD2-positive tumor cells were classified as negative. No additional numerical cutoff was applied beyond the requirement for unequivocal identification of GD2-positive neuroblastoma cells. Because this was a retrospective study, no prospectively predefined adjudication algorithm was available for discordant results between GD2-directed immunocytochemistry and flow cytometry. Accordingly, the GD2-based classification used in the present analysis should be regarded as a study reference classification rather than an absolute diagnostic gold standard. Conventional bone marrow cytomorphology was used to assess overt marrow involvement and to apply the study-specific exclusion criterion. Imaging modalities, including MIBG scintigraphy and PET/CT, are important for systemic staging and evaluation of metastatic disease but were not incorporated into the reference classification for the present bone marrow cell-level endpoint. Molecular assays, including QRT-PCR, represent additional sensitive approaches for detecting minimal neuroblastoma cells; however, the present study did not perform a systematic head-to-head comparison between GD2-based classification and molecular or imaging modalities. Consequently, the reference classification was restricted to the available institutional GD2-based assessments, and potential misclassification cannot be excluded.

The analytical sensitivity and specificity of the GD2-based assays were not independently re-estimated in the present cohort. Therefore, no new assay-performance estimates are claimed in this study, and previously published methodological information is cited separately where applicable.

### Peripheral blood sample analysis

Routine peripheral blood parameters were detected using a Sysmex XN-1000 automated hematology analyzer with matched supporting reagents. Coagulation indices were measured by a Sysmex CS5100 automated coagulation analyzer. Serum biochemical indicators and tumor markers were determined using a Mindray BS2800-CL8000i fully automatic biochemical and immunoassay analyzer with corresponding reagents.

### Immunocytometric analysis of bone marrow aspiration samples in high-risk NB patients

For the exploratory analysis of monocyte-macrophage polarization, bone marrow samples from 20 patients were included, comprising 10 patients with GD2-positive bone marrow involvement and 10 patients with GD2-negative bone marrow findings. Bone marrow nucleated cells were obtained by density gradient centrifugation. Antibodies for detection of M2-like monocyte-macrophage cells included CD206-FITC antibody (clone no. SK7), CD86-APC antibody (clone no. SK1), CD14-PerCP antibody (clone no. 2D1). CD14^+^ CD206^+^ cells were operationally defined as monocyte-macrophages with an M2-associated phenotype for the purposes of this study. CD206 was used as an M2-associated marker rather than as a definitive marker of a homogeneous M2 macrophage population. For the ROC analysis evaluating the discriminatory performance of CD14^+^ CD206^+^ monocyte-macrophages, 30 patients with complete flow-cytometric measurements and reference MRD classification were included. The ROC analysis was restricted to patients for whom complete CD14^+^CD206^+^ flow-cytometric data were available.

### Statistical analysis

All statistical analyses were performed using SPSS version 18.0 (IBM Corp., Armonk, NY, USA) and GraphPad Prism version 10.1.2 (GraphPad Software, Boston, MA, USA). Continuous variables are presented as mean ± standard deviation (SD), unless otherwise specified. A two-sided P value <0.05 was considered statistically significant.

Receiver operating characteristic (ROC) curve analysis was performed to evaluate the ability of laboratory parameters to discriminate between MRD-positive and MRD-negative patients. For individual biomarkers, empirical non-parametric ROC curves were constructed directly from the observed measurements. The area under the ROC curve (AUC) was calculated using the trapezoidal method, and its standard error and 95% confidence interval (CI) were estimated using the non-parametric DeLong method.

To evaluate the combined discriminatory performance of routinely available peripheral blood biomarkers, a multivariable logistic regression model was constructed using NSE, hemoglobin, monocyte count, C-reactive protein, fibrinogen, and D-dimer as predictor variables, with MRD status as the binary dependent variable. The predicted probabilities generated by this model were used to construct the combined empirical ROC curve. The AUC and its 95% CI were calculated using the same methodology. The optimal cutoff was determined by maximizing the Youden index (sensitivity + specificity − 1), and the corresponding sensitivity and specificity were calculated.

## Results

### Peripheral blood clinicopathological features in NB patients with positive bone marrow MRD

This study analyzed peripheral blood routine data from 143 patients pathologically diagnosed with neuroblastoma (NB). In MRD-positive patients, the white blood cell (WBC) count was (5.17 ± 2.70)×10^9^/L, monocyte count was (0.36 ± 0.26)×10^9^/L, and C-reactive protein (CRP) level was (29.36 ± 36.99) mg/L (**Table 1**). Their plasma fibrinogen was 3.95 ± 1.61 g/L, and D-dimer was 3411.60 ± 3087.57 μg/L (**Table 2**). Levels of neuron-specific enolase (NSE), lactate dehydrogenase (LDH) and transferrin were 263.30 ± 417.65 μg/mL, 645.93 ± 1179.25 U/L and 687.34 ± 867.73 μg/L, respectively (**Table 3**). For MRD-negative patients, WBC count was (6.93 ± 5.41)×10^9^/L, monocyte count was (0.88 ± 1.98)×10^9^/L, and CRP was (17.11 ± 36.11) mg/L (**Table 1**). Plasma fibrinogen was 3.02 ± 1.59 g/L, and D-dimer was 2207.98 ± 2833.88 μg/L (**Table 2**). NSE, LDH and transferrin levels were 102.33 ± 216.70 μg/mL, 484.62 ± 135.79 U/L and 398.90 ± 420.19 μg/L (**Table 3**). Compared with MRD-negative patients, MRD-positive patients exhibited significantly lower WBC and monocyte counts, accompanied by elevated levels of CRP, fibrinogen, D-dimer, NSE, LDH and transferrin.

**Table 1:** Peripheral blood clinicopathological features of NB patients.

Laboratory indicators	MRD^+^ (x ± SD)	MRD^-^ (x ± SD)	*p*
HGB (g/L)	95.74 ± 24.07	104.88 ± 29.97	0.055
WBC (*10^9^/L)	5.17 ± 2.70	6.93 ± 5.41	0.012*
Neutrophils (*10^9^/L)	2.90 ± 1.81	4.95 ± 9.72	0.253
Lymphocytes (*10^9^/L)	1.85 ± 1.20	2.01 ± 1.72	0.054
Monocytes (*10^9^/L)	0.36 ± 0.26	0.88 ± 1.98	0.015*
PLT (*10^9^/L)	229.38 ± 146.82	259.42 ± 142.68	0.065
NLR	3.00 ± 4.87	10.54 ± 64.96	0.233
PLR	368.18 ± 1051.35	358.79 ± 740.15	0.278
CRP (mg/L)	29.36 ± 36.99	17.11 ± 36.11	0.037*

* *p* < 0.05, * *p < 0.01, Bold values indicate statistically significant P-values (*p* < 0.05).

**Table 2:** Blood coagulation features in NB patients.

Laboratory indicators	MRD^+^ (x ± SD)	MRD^-^ (x ± SD)	*p*
Fibrinogen (g/L)	3.95 ± 1.61	3.02 ± 1.59	0.045*
AT3 (%)	98.44 ± 25.67	94.90 ± 19.98	0.341
D-dimer (mg/L)	3411.60 ± 3087.57	2207.98 ± 2833.88	0.018*

* *p* < 0.05, * *p < 0.01, Bold values indicate statistically significant P-values (*p* < 0.05).

**Table 3:** Peripheral blood biochemistry and tumor markers of NB patients.

Laboratory indicators	MRD^+^ (x ± SD)	MRD^-^ (x ± SD)	*p*
NSE (ug/L)	263.30 ± 417.65	102.33 ± 216.75	0.011*
LDH (U/L)	645.93 ± 1179.25	484.62 ± 135.79	0.048*
Ferr (ug/L)	687.34 ± 867.73	398.90 ± 420.19	0.008**

LDH, Lactic dehydrogenase; NSE, Neuron-specific enolase; Ferr Ferritin.

* *p* < 0.05, * *p < 0.01, Bold values indicate statistically significant P-values (*p* < 0.05).

### Diagnostic performance of serological markers for bone marrow MRD positivity in NB

We utilized ROC curve analysis to evaluate the diagnostic performance of differentially expressed peripheral blood biomarkers. Only NSE, HGB, monocytes, CRP, fibrinogen and D-dimer exhibited statistically significant diagnostic potential (AUC: 0.615–0.677, all P<0.05). Since all AUC values were less than 0.7, these markers lacked satisfactory standalone accuracy for clinically diagnosing bone marrow MRD positivity (**Figures 1a–f**).

**Figure 1:**
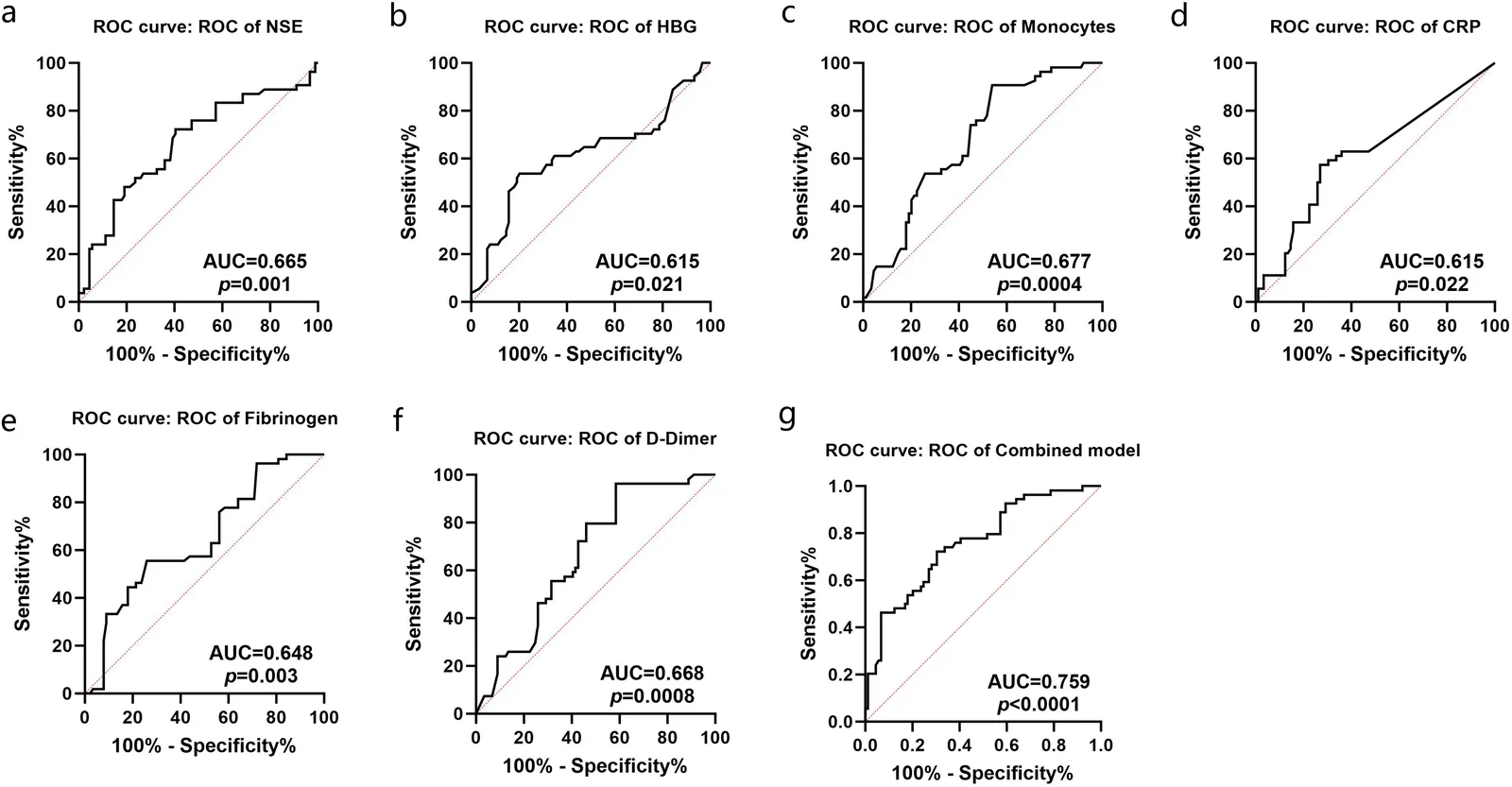
ROC analysis of different serological indicators for identifying patients with MRD-positive bone marrow. **(a)** Peripheral blood NSE; **(b)** Hemoglobin; **(c)** Monocytes; **(d)** CRP; **(e)** Plasma fibrinogen; **(f)** D-dimer; **(g)** ROC curve of the combined peripheral blood biomarker model incorporating NSE, hemoglobin, monocyte count, CRP, fibrinogen, and D-dimer (AUC = 0.759, 95% CI 0.678–0.840).

To determine whether integration of peripheral blood biomarkers could improve discrimination of bone marrow MRD status, we constructed a multivariable logistic regression model incorporating NSE, hemoglobin, monocyte count, CRP, fibrinogen, and D-dimer. Individual biomarkers showed modest diagnostic performance, with AUCs ranging from 0.615 to 0.677. In contrast, the combined model achieved an AUC of 0.759 (95% CI, 0.678–0.840). At the optimal predicted-probability cutoff of 0.365, the sensitivity and specificity were 72.2% and 69.7%, respectively (**Table 4**; **Figure 1g**). The combined model showed a higher AUC than any individual biomarker in the present cohort, although its overall discriminatory performance remained moderate. The incremental value of combining these markers requires further validation.

**Table 4:** Diagnostic performance of individual and combined peripheral blood biomarkers for bone marrow MRD in high-risk neuroblastoma.

Peripheral blood biomarker/model	AUC	95% CI	Optimal cutoff	Sensitivity (%)	Specificity (%)
NSE	0.665	0.569–0.760	≥30.9	72.2	59.6
HGB	0.615	0.513–0.717	≤86	53.7	79.8
Monocyte count	0.677	0.590–0.765	≤0.54	90.7	46.1
CRP	0.615	0.522–0.708	≥11.05	57.4	73
Fibrinogen	0.649	0.556–0.741	≥3.606	55.6	74.2
D-dimer	0.668	0.580–0.756	≥362.17	96.3	41.6
Combined model	0.759	0.678–0.840	Predicted probability ≥0.365	72.2	69.7

### Association between GD2-positive bone marrow involvement and polarization phenotypes of CD14^+^ monocyte-macrophages

Subsequently, bone marrow samples from 10 NB patients complicated with bone marrow metastasis (GD2-positive) and another 10 NB patients without bone marrow metastasis (GD2-negative) were collected. Flow cytometry was adopted to detect changes in bone marrow nucleated cells, especially monocyte-macrophages. A stepwise gating strategy of flow cytometry was utilized to isolate bone marrow CD14^+^ monocyte-macrophages, and phenotypic differences of macrophage polarization were compared between GD2-negative (GD2^−^) and GD2-positive (GD2^+^) neuroblastoma groups, followed by correlation validation using clinical samples.

Flow cytometry gating results (**Figure 2a**) showed that there was no statistically significant difference in the absolute count of bone marrow CD14^+^ monocyte-macrophages between the GD2^−^ group and GD2^+^ group (NS, P > 0.05; **Figure 2d**), indicating that the overall absolute number of bone marrow CD14^+^ monocyte-macrophages did not differ significantly between the GD2-positive and GD2-negative groups. Quantitative results of polarized subsets illustrated that the percentage and absolute quantity of CD14^+^CD86^+^M1-associated monocyte-macrophages and CD14^+^CD206^+^ CD14^+^CD206^+^M2-like monocyte-macrophages were significantly higher in the GD2^+^ group than those in the GD2^−^ group (***, P < 0.001). The single-parameter histograms of CD86 and CD206 displayed a marked right shift of fluorescence intensity in the GD2^+^ group (**Figure 2c**). Notably, the increase was more pronounced for the CD14^+^CD206^+^ subset than for the CD14^+^CD86^+^ subset.

**Figure 2:**
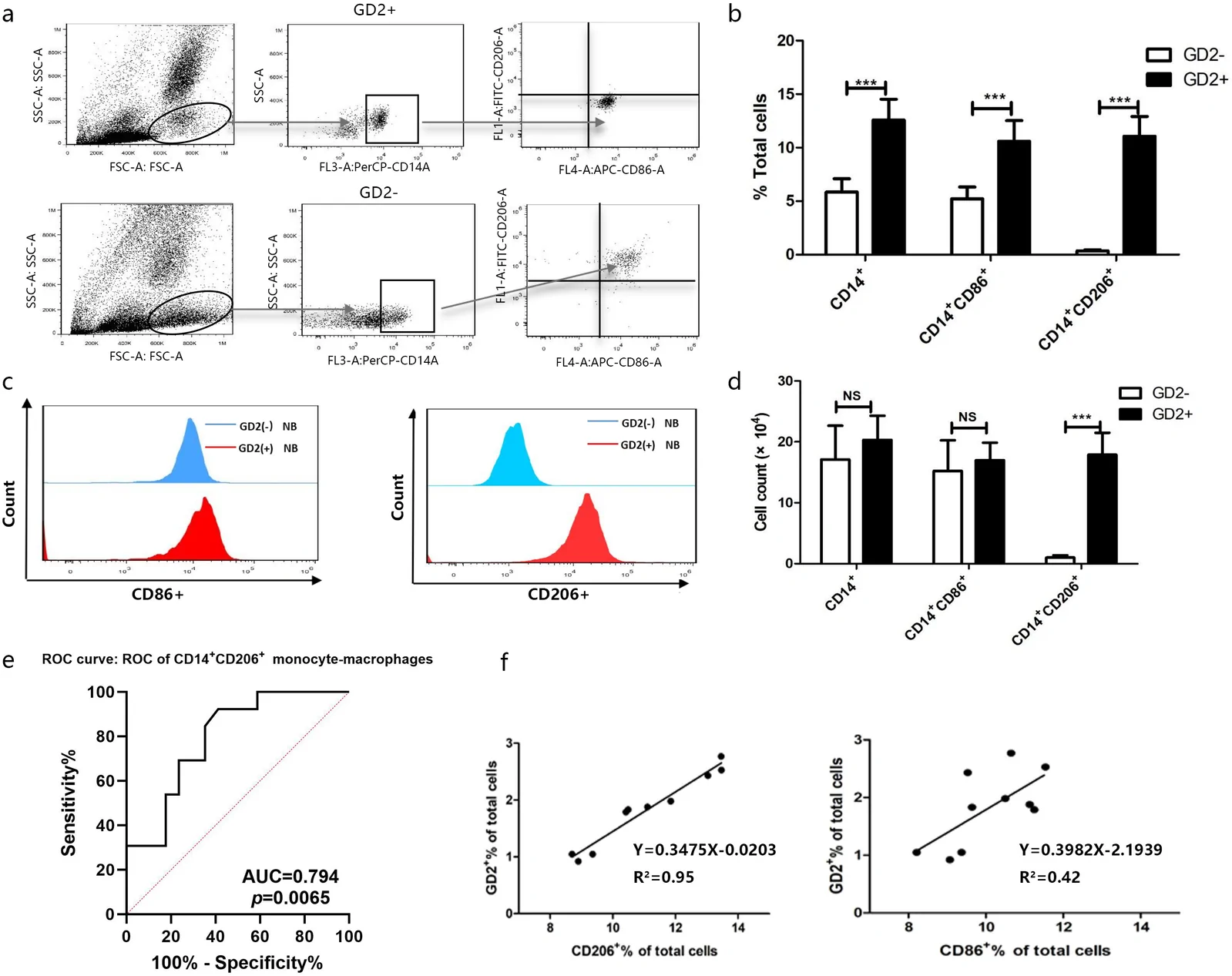
Association of GD2-positive bone marrow involvement with CD14^+^ monocyte-macrophage polarization phenotypes and their discriminatory performance for bone marrow MRD **(a)** Representative flow-cytometric gating strategy for identifying CD14^+^ monocyte-macrophages and their M1/M2-associated phenotypic subsets in bone marrow samples from GD2-negative and GD2-positive patients. The polarization analysis included 20 patients (10 GD2-positive and 10 GD2-negative). **(b)** Bar graph presenting the percentage of CD14^+^ total monocyte-macrophages, CD14^+^CD86^+^ M1-associated monocyte-macrophages and CD14^+^CD206^+^ CD14^+^CD206^+^ M2-like monocyte-macrophages relative to total bone marrow nucleated cells. **(c)** Single-parameter fluorescence intensity histograms of CD86 (M1 marker) and CD206 (M2-associated marker) in CD14^+^ monocyte-macrophages; blue curves = GD2^−^ group, red curves = GD2^+^ group. **(d)** Absolute cell counts (×10^4^) of CD14^+^, CD14^+^CD86^+^ and CD14^+^CD206^+^ macrophage subsets. NS = no significant difference; ***P < 0.001 versus GD2^−^ group. **(e)** ROC curve evaluating the diagnostic value of CD14^+^CD206^+^ monocyte-macrophage count for bone marrow minimal residual disease (MRD) in 30 high-risk NB patients; AUC = 0.794,*p* = 0.0065. (n=30) **(f)** Linear regression correlation scatter plots between the proportion of bone marrow GD2^+^ tumor cells and the percentage of polarized macrophage subsets. Left: CD14^+^CD206^+^ macrophages (Y = 0.3475X−0.0203, R²=0.95); Right: CD14^+^CD86^+^ macrophages (Y = 0.3982X−2.1939, R²=0.42). The flow-cytometric phenotypic analyses, including the between-group comparisons and correlation analyses, were performed using bone marrow samples from 20 patients (GD2-positive, n = 10; GD2-negative, n = 10). Panels **(a–d, f)** present data from this 20-patient cohort. The ROC analysis shown in panel **(e)** was performed using a separate analytical dataset comprising 30 patients with complete flow-cytometric measurements and GD2-based reference MRD classification (n = 30). All sample numbers refer to independent biological samples from individual patients.

Clinical correlation analysis showed a strong positive association between the proportion of GD2-positive tumor cells in bone marrow and the percentage of CD14^+^CD206^+^ monocyte-macrophages (Y = 0.3475X − 0.0203, R² = 0.95). A less pronounced positive association was observed for CD14^+^CD86^+^ monocyte-macrophages (Y = 0.3982X − 2.1939, R² = 0.42; **Figure 2f**). These results describe associations within the analyzed clinical samples and do not establish a direct regulatory effect of GD2 on macrophage polarization. These findings indicate a positive association between the proportion of GD2-positive tumor cells and the abundance of CD14^+^CD206^+^ monocyte-macrophages in the analyzed bone marrow samples, without establishing a causal relationship.

### Discriminatory performance of CD14^+^CD206^+^ monocyte-macrophages for bone marrow MRD status

ROC curve analysis was performed on bone marrow specimens from 30 children diagnosed with high-risk neuroblastoma to evaluate the diagnostic performance of CD14^+^CD206^+^ monocyte-macrophages for bone marrow minimal residual disease (MRD) (**Figure 2e**). The area under the ROC curve (AUC) was 0.794 (P = 0.0065), with an optimal cutoff value of 1.71 × 10^4^/mL, indicating moderate discriminatory performance in this exploratory cohort. These findings suggest that CD14^+^CD206^+^ monocyte-macrophages may have potential as an auxiliary immune biomarker for bone marrow MRD assessment in high-risk NB.

In summary, GD2-positive bone marrow involvement was not associated with a significant change in the overall abundance of CD14^+^ monocyte-macrophages, but was associated with higher proportions and absolute counts of polarized CD14^+^CD86^+^ and CD14^+^CD206^+^ subsets. The CD14^+^CD206^+^ population showed a particularly strong positive correlation with the proportion of GD2-positive tumor cells and demonstrated moderate discriminatory performance for bone marrow MRD status. These findings support further evaluation of CD14^+^CD206^+^ monocyte-macrophages as a potential auxiliary immune biomarker associated with the bone marrow tumor microenvironment.

## Discussion

In the present study, we evaluated peripheral blood biomarkers and bone marrow immune-cell phenotypes in high-risk neuroblastoma (NB) with respect to bone marrow minimal residual disease (MRD). Individual peripheral blood parameters showed limited discriminatory performance, whereas a multivariable model integrating NSE, hemoglobin, monocyte count, C-reactive protein, fibrinogen, and D-dimer achieved moderate discrimination (AUC 0.759, 95% CI 0.678–0.840). More importantly, bone marrow CD14^+^CD206^+^ monocyte-macrophages were increased in patients with GD2-positive bone marrow involvement and showed a strong association with the proportion of GD2-positive tumor cells. In the 30-patient ROC cohort, CD14^+^CD206^+^ monocyte-macrophages showed an AUC of 0.794, with an optimal cutoff of 1.7×10^4^/mL. These findings provide preliminary evidence that a bone marrow immune-microenvironmental phenotype may contain information associated with low-level tumor involvement, although the diagnostic value of this marker requires further validation.

The interpretation of CD14^+^CD206^+^ cells also requires careful consideration of macrophage biology. Macrophage activation is now understood as a phenotypic continuum rather than a strict M1/M2 dichotomy (24). CD206 is widely used as an M2-associated marker, but CD206 expression alone is insufficient to define a homogeneous or functionally validated M2 macrophage population (25, 26). Therefore, in the present study, the term “M2-like” is used to describe CD14^+^CD206^+^ monocyte-macrophages with an M2-associated phenotype, rather than to imply a definitive functional M2 state. Future studies incorporating additional phenotypic and functional markers, such as CD163, ARG1, IL-10, or transcriptomic profiling, will be required to more comprehensively characterize this population.

Contemporary reviews of tumor-associated macrophages similarly emphasize spatial, developmental, and functional diversity beyond the conventional M1/M2 classification. Accordingly, CD206 positivity should be treated as an operational phenotypic readout rather than proof of a stable, suppressive macrophage state (27, 28).

Our findings are also consistent with the concept that neuroblastoma bone marrow involvement is accompanied by remodeling of the local immune microenvironment. Previous clinical studies have reported increased tumor-associated inflammatory-cell and macrophage infiltration in metastatic neuroblastoma, supporting an association between metastatic disease and an altered immune microenvironment (29). More recently, single-cell transcriptomic and epigenomic profiling of neuroblastoma bone marrow metastases demonstrated extensive interactions between neuroblastoma cells and bone marrow myeloid cells. In that study, monocytes exhibited both M1- and M2-associated features and expressed tumor-promoting programs, providing molecular evidence that the metastatic niche is actively remodeled during bone marrow involvement (30). These observations provide a biological context for our finding that CD14^+^CD206^+^ monocyte-macrophages are strongly associated with GD2-positive tumor-cell involvement. Furthermore, the strong correlation does not establish that CD14^+^CD206^+^ assessment provides diagnostic information independent of GD2-positive tumor-cell burden. Whether this immune-cell phenotype contributes additional diagnostic or prognostic information beyond direct tumor-cell detection remains to be determined. Importantly, the observational nature of the present study precludes causal inference. Although the abundance of CD14^+^CD206^+^ monocyte-macrophages was positively associated with GD2-positive tumor-cell burden, these findings do not demonstrate that GD2 itself directly induces or regulates M2-like macrophage polarization. The observed association may reflect broader changes in the bone marrow immune microenvironment accompanying neuroblastoma involvement. Functional studies, including tumor cell–macrophage co-culture experiments and targeted perturbation of relevant signaling pathways, will be required to investigate the underlying mechanisms and establish whether a causal relationship exists.

Peripheral blood biomarkers and bone marrow immune-cell phenotypes may reflect different biological dimensions of disease. NSE is an established neuroblastoma biomarker with recognized prognostic value, but its specificity for low-level bone marrow disease is limited (12). Similarly, serum LDH and ferritin have established prognostic associations with high-risk neuroblastoma, whereas their ability to discriminate low-level bone marrow disease in our cohort was limited. The combined peripheral blood model showed a higher AUC than the individual parameters in the present cohort, although its overall discriminatory performance remained moderate. The incremental diagnostic value of combining these systemic indicators requires further validation. These findings support the distinction between systemic disease-associated biomarkers and direct or local measurements of the bone marrow metastatic niche.

From a biological perspective, CD14^+^CD206^+^ assessment captures a cellular phenotype within the bone marrow microenvironment rather than directly measuring tumor-cell transcripts or genomic abnormalities. Flow cytometry can simultaneously characterize surface phenotypes at the single-cell level and quantify multiple cellular subsets within the same specimen. This may provide complementary biological information to molecular measurements of tumor burden. However, the present study does not establish that protein-level assessment is inherently superior to transcript-based or other molecular approaches, and no direct comparison of analytical performance, cost, turnaround time, or technical complexity was performed. Thus, the potential value of CD14^+^CD206^+^ assessment should be considered complementary to, rather than a replacement for, established tumor-cell detection approaches.

From a clinical perspective, GD2-based assays and CD14^+^CD206^+^ assessment provide fundamentally different types of information. GD2-directed immunocytochemistry and GD2-based flow cytometry are established cell-based approaches for detecting neuroblastoma cells in bone marrow and are consistent with the broader International Neuroblastoma Risk Group framework for sensitive detection of disseminated neuroblastoma cells (31). In the present study, these GD2-based approaches were used within the institutional workflow to establish the study reference classification. We therefore consider CD14^+^CD206^+^ monocyte-macrophages to be a complementary immune-microenvironmental marker rather than an alternative to direct tumor-cell detection. Both approaches require an adequate bone marrow specimen, and the present study did not prospectively compare them with respect to turnaround time, cost, required cell number, technical complexity, or assay failure rate. Consequently, no operational or economic advantage of CD14^+^CD206^+^ assessment over GD2-based methods can be inferred from the current data. One potential setting for future investigation is the concurrent assessment of GD2-positive tumor-cell burden and CD14^+^CD206^+^ immune-cell phenotypes in patients undergoing clinically indicated bone marrow evaluation. Such an approach could allow tumor-cell involvement and the accompanying immune-microenvironmental state to be characterized using material obtained during the same aspiration procedure. However, whether this additional immune-cell information improves clinical interpretation, treatment monitoring, or patient management beyond GD2-based detection has not been established.

Several limitations should be considered when interpreting these findings. First, the flow-cytometric polarization analysis included only 20 patients (10 GD2-positive and 10 GD2-negative), and the ROC analysis included 30 patients. Because neuroblastoma is a relatively uncommon pediatric malignancy and the present analysis required high-risk disease, adequate bone marrow specimens, and complete flow-cytometric and reference-classification data, the available exploratory cohort was limited. The estimated effect sizes, correlation coefficients, AUC, and proposed cutoff may therefore be sensitive to sampling variation, limiting reproducibility and generalizability. Larger, independent cohorts will be needed to obtain more precise estimates of diagnostic performance, assess the stability of the proposed cutoff, and establish the reproducibility and generalizability of these findings. Second, potential confounding by established clinical and biological risk factors could not be adequately addressed. Although patient age was available and all participants were classified as having high-risk neuroblastoma, the exploratory CD14^+^CD206^+^ ROC analysis included only 30 patients. Comprehensive information on MYCN amplification, 11q and 1p chromosomal abnormalities, image-defined risk factors, primary tumor site, and other relevant disease characteristics was not systematically available for this subgroup. Furthermore, variation in tumor burden and metastatic disease characteristics may influence both MRD status and the bone marrow immune-cell composition. Given the limited sample size and incomplete risk-factor information, a reliable multivariable adjustment or stratified analysis could not be performed. Therefore, residual confounding cannot be excluded, and the observed association does not establish that CD14^+^CD206^+^ monocyte-macrophages are independently associated with bone marrow MRD after adjustment for established clinical and genomic risk factors. Therefore, a reliable multivariable adjustment or MYCN-stratified analysis could not be performed without substantial missing-data bias and loss of precision. Consequently, residual confounding cannot be excluded, and the present findings do not establish that the discriminatory performance of CD14^+^CD206^+^ monocyte-macrophages is independent of established clinical and genomic risk factors. Third, GD2-directed immunocytochemistry and GD2-based flow cytometry were used within our institutional workflow to establish the study reference classification rather than an absolute diagnostic gold standard. The analytical sensitivity and specificity of these assays were not independently established in the present cohort. Furthermore, because no prospectively predefined adjudication algorithm was available for discordant immunocytochemical and flow-cytometric findings, potential misclassification of bone marrow disease status cannot be excluded. These limitations should be considered when interpreting the estimated discriminatory performance of CD14^+^CD206^+^ monocyte-macrophages. Finally, the study was conducted at a single center and lacks independent external validation.In addition, we did not perform a formal incremental-value analysis to determine whether incorporating CD14^+^CD206^+^ measurements into GD2-based assessment improves diagnostic discrimination or clinical decision-making. Larger prospective, multicenter cohorts with comprehensive clinical and genomic annotation will be required to determine the incremental diagnostic value and clinical applicability of CD14^+^CD206^+^ assessment.

Taken together, our findings indicate that bone marrow CD14^+^CD206^+^ M2-like monocyte-macrophages are associated with GD2-positive tumor-cell involvement and show moderate discriminatory performance for bone marrow MRD in high-risk neuroblastoma. The findings support the potential role of this population as a complementary immune-microenvironmental biomarker, rather than as a replacement for direct GD2-based tumor-cell detection. Because the present data are exploratory and observational, they do not establish causality, statistical independence from established risk factors, or definitive clinical utility. Larger prospective and independent validation studies will be necessary to determine whether CD14^+^CD206^+^ assessment provides clinically meaningful incremental information beyond established methods for bone marrow MRD detection.

## Data Availability

The raw data supporting the conclusions of this article will be made available by the authors, without undue reservation.
